# The Writers, Readers, and Erasers in Redox Regulation of GAPDH

**DOI:** 10.3390/antiox9121288

**Published:** 2020-12-16

**Authors:** Maria-Armineh Tossounian, Bruce Zhang, Ivan Gout

**Affiliations:** Department of Structural and Molecular Biology, University College London, London WC1E 6BT, UK; m.tossounian@ucl.ac.uk (M.-A.T.); bruce.zhang.18@ucl.ac.uk (B.Z.)

**Keywords:** GAPDH, metabolism, moonlighting protein, redox regulation, oxidative PTMs, *S*-thiolation

## Abstract

Glyceraldehyde 3–phosphate dehydrogenase (GAPDH) is a key glycolytic enzyme, which is crucial for the breakdown of glucose to provide cellular energy. Over the past decade, GAPDH has been reported to be one of the most prominent cellular targets of post-translational modifications (PTMs), which divert GAPDH toward different non-glycolytic functions. Hence, it is termed a moonlighting protein. During metabolic and oxidative stress, GAPDH is a target of different oxidative PTMs (oxPTM), e.g., sulfenylation, *S*-thiolation, nitrosylation, and sulfhydration. These modifications alter the enzyme’s conformation, subcellular localization, and regulatory interactions with downstream partners, which impact its glycolytic and non-glycolytic functions. In this review, we discuss the redox regulation of GAPDH by different redox writers, which introduce the oxPTM code on GAPDH to instruct a redox response; the GAPDH readers, which decipher the oxPTM code through regulatory interactions and coordinate cellular response via the formation of multi-enzyme signaling complexes; and the redox erasers, which are the reducing systems that regenerate the GAPDH catalytic activity. Human pathologies associated with the oxidation-induced dysregulation of GAPDH are also discussed, featuring the importance of the redox regulation of GAPDH in neurodegeneration and metabolic disorders.

## 1. Introduction

**Figure 1:**
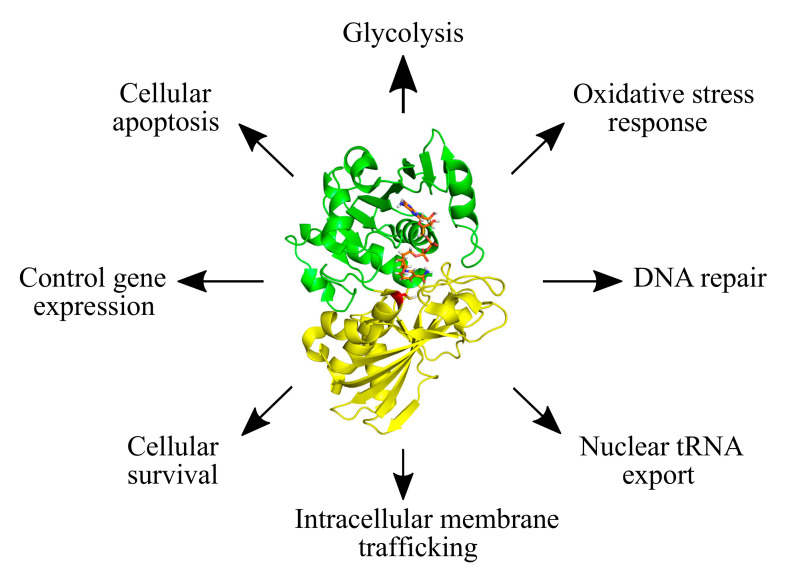
The diversity of cellular functions of GAPDH. Glycolytic and non-glycolytic functions of GAPDH are shown. The structure of GAPDH is featured (PDB ID: 4WNC) with its N-terminal NAD^+^-binding domain indicated in green, and the C-terminal catalytic domain in yellow. The catalytic cysteine residue is shown in red and the NAD^+^ molecule in orange.

Glyceraldehyde-3-phosphate dehydrogenase (GAPDH) is one of the most abundant proteins, well known for its function in cellular metabolism. During glycolysis, GAPDH catalyzes the oxidative phosphorylation of glyceraldehyde-3-phosphate (G3P) to glycerate-1,3-bisphosphate, with the concomitant release of reduced nicotinamide adenine dinucleotide (NADH) [1,2]. The important role of GAPDH in cellular metabolism has led to extensive functional and regulatory studies, which have also uncovered diverse non-glycolytic functions (Figure 1) [3,4,5,6,7,8,9]. Many of these functions are controlled by different post-translational modifications (PTMs) on GAPDH, e.g., phosphorylation, acetylation, sulfenylation, nitrosylation, sulfhydration, and *S*-thiolation, among others [8,10,11,12,13,14,15,16,17,18,19]. These PTMs impact the enzymatic activity of GAPDH, its regulatory interactions and its subcellular localization [8,10,11,12,13,14,15,16,17,18,19].

The regulation of GAPDH by oxidative PTMs (oxPTMs, e.g., sulfenylation, disulfide bond formation, nitrosylation, sulfhydration, glutathionylation, and other *S*-thiolations) has been the subject of extensive studies since the beginning of this century [8,17,18,20]. The catalytic cysteine of GAPDH plays a central role in the redox regulation of GAPDH. Different oxPTMs introduced on the catalytic cysteine inhibit the glycolytic function of GAPDH, causing the disruption of glycolysis. This can lead to rerouting of the cellular carbohydrate flux toward the pentose phosphate shunt, which generates NADPH+H^+^ [21,22]. The latter plays an important role as the final electron donor to different cellular reducing pathways that have a key role in reestablishing and maintaining the cellular reducing environment under stress conditions [22,23]. In this review, we present an overview of the redox regulation of GAPDH by the different redox writers (e.g., hydrogen peroxide). We will also shed light on the consequences of the introduction of oxPTMs on GAPDH and the role of readers and erasers in the redox regulation of its subcellular localization and functional interactions with downstream partners. Finally, we will discuss the different human pathologies (e.g., neurodegeneration and metabolic disorders) associated with the oxidation-induced dysregulation of GAPDH.

## 2. GAPDH Modifications by Redox Writers

### 2.1. Key Features of Redox Writers

Redox homeostasis is achieved and maintained by the coordinated control of redox reactive species (RRS) production and removal. RRS, such as reactive oxygen (ROS), nitrogen (RNS) and sulfur (RSS) species constitute groups of cellular metabolites, which contribute to the redox balance of the cell. ROS are produced endogenously as by-products of cellular respiration and exhibit a dual role: beneficial or harmful to cells. Under physiological conditions, ROS play an important role in cellular signaling and metabolic pathways. However, prolonged exposure of cells to elevated levels of ROS, caused by environmental variations, metabolic changes or disease [24,25], may lead to the damage of DNA, lipids and proteins [26,27]. ROS can be generated via electron-based redox cycling, and can be in the form of a radical (e.g., hydroxyl radical, superoxide anion) or non-radical (e.g., hydrogen peroxide (H_2_O_2_)). These oxidants, referred to as redox writers in this review, modify target proteins by priming oxPTMs, which can trigger further redox-associated intra- or intermolecular modifications and interactions.

**Figure 2:**
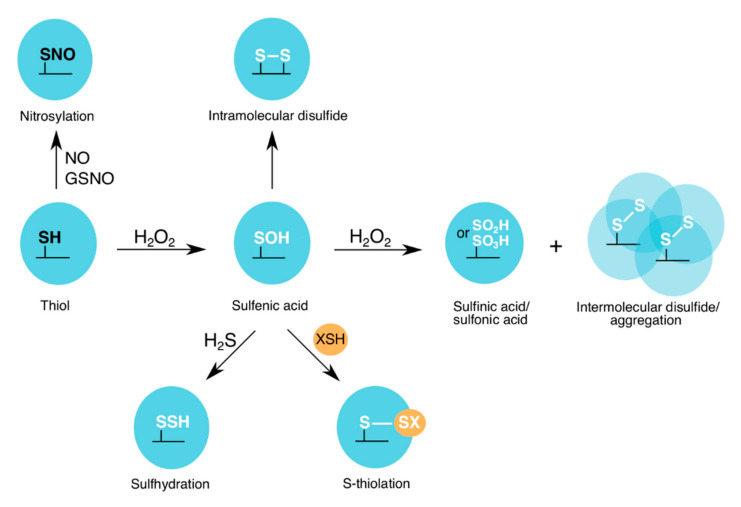
oxPTMs on the catalytic cysteine of GAPDH. At lower levels of H_2_O_2_, a sulfenic acid is formed on the GAPDH catalytic cysteine. However, at higher H_2_O_2_ levels, sulfinic/sulfonic acids and intermolecular disulfides are formed. The latter can lead to GAPDH aggregation. To prevent further cysteine overoxidation, either intramolecular disulfides or mixed disulfides with LMW thiols (e.g., glutathione (GSH), bacillithiol (BSH), mycothiol (MSH) and coenzyme A (CoASH)) are formed. GAPDHs from different organisms have been shown to be nitrosylated or sulfhydrated by nitric oxide (NO) and nitroso-glutathione (GSNO), or hydrogen sulfide (H_2_S), respectively.

Under oxidizing conditions, such as H_2_O_2_-induced stress, the sulfur-containing cysteine residues are most susceptible to oxidation. At lower levels of H_2_O_2_ (physiological conditions (“oxidative eustress”), 1–10 nM) [28], protein cysteine thiols, which have a high nucleophilic character, are oxidized to a sulfenic acid state (reversible oxPTM) [29]. Higher levels of H_2_O_2_ (supraphysiological conditions (“oxidative distress”), >100 nM) [28] can cause further oxidation of the cysteine to a sulfinic acid (mostly irreversible oxPTM) or a sulfonic acid (irreversible oxPTM) state (Figure 2) [29]. To prevent cysteine overoxidation and loss of protein function, cysteine residues can form intra- or intermolecular disulfide bonds, as well as mixed disulfides with low molecular weight (LMW) thiols (Figure 2). Formation of a mixed disulfide bond on a protein is termed thiolation, which “edits” the sulfenic acid and creates new redox-induced binding motifs. These motifs are recognized by the “readers”, which promote redox signaling and antioxidant response. Reduction of the modified cysteine residues is mediated by specific antioxidant recycling enzymes.

In addition to ROS, during cellular stress, other types of redox writers are also generated, and these include: (a) RNS, such as nitric oxide (NO) and peroxynitrite; and (b) RSS, such as hydrogen sulfide (H_2_S) [30,31]. Both species are capable of modifying cysteine residues by nitrosylation or sulfhydration, respectively (Figure 2). Overall, once the redox writers (ROS, RNS and RSS) have primed the protein by introducing an “oxPTM code”, as part of the “Redox Code” [32], e.g., sulfenylation, nitrosylation or sulfhydration on a target cysteine, the protein’s activity, interaction with downstream partners and subcellular localization can be altered.

### 2.2. Redox Sensing by GAPDH and Its Regulatory Consequences

GAPDH is a homo-tetramer, with each monomer composed of two domains: the N-terminal NAD^+^-binding domain, which displays the characteristic Rossmann fold [33]; and the C-terminal catalytic domain, which carries the conserved catalytic cysteine (Figure 1) [34,35,36,37]. During catalysis, the GAPDH catalytic cysteine binds to its substrate (G3P), through a thiohemiacetal intermediate formation. A histidine residue, located in the microenvironment of the active site, facilitates this reaction by lowering the *pKa* of the catalytic cysteine, leading to its deprotonation, followed by its interaction with G3P [38,39]. During oxidative stress, the redox-sensitive catalytic cysteine of GAPDH is modified by different oxPTMs [8,17,18,40], e.g., sulfenylation, disulfide bond formation, nitrosylation, sulfhydration, glutathionylation and other forms of *S*-thiolation, which lead to changes in its catalytic activity and glycolytic function [23,41] (Figure 2, Table 1; Table 2).

#### 2.2.1. Sulfenylation and S-Thiolation of GAPDH

GAPDH is a highly abundant cellular protein, known to be one of the most prominent targets of H_2_O_2_ during cellular stress [18,42,43]. Global proteome analyses of oxidative stress-induced PTMs in different organisms have identified the catalytic cysteine of GAPDH homologs as the most abundant targets of both sulfenylation and *S*-thiolation [20,40,44,45,46,47,48,49,50]. Using genetic (e.g., YAP1C probe) and chemical (e.g., DYn-2 and BTD probes) approaches, combined with mass spectrometric analyses, the *Arabidopsis thaliana* and *Homo sapiens* sulfenomes (sulfenylated proteins) were identified [49,50,51,52,53]. Sulfenylation of GAPDH was reported, in addition to other proteins with important metabolic functions. Further in vitro studies showed that H_2_O_2_ oxidizes the GAPDH catalytic cysteine to a sulfenic acid [23,41,54,55], and depending on the GAPDH homolog, this reaction occurs with a second order rate constant of 10–10^3^ M^−1^s^−1^ [41,55,56]. Subsequently, the sulfenic acid can form intra- or intermolecular disulfide bonds, as well as mixed-disulfides with LMW thiols (Figure 2, Table 1 and Table 2). An in vitro study performed by Nakajima et al. (2007) showed that the rabbit muscle GAPDH undergoes conformational changes in the presence of oxidizing agents (e.g., nitric oxide) [57]. These conformational changes facilitate the formation of an intermolecular disulfide bond between two catalytic cysteine residues of GAPDH [57]. The latter induces further conformational changes, which expose non-catalytic cysteine residues. The exposed cysteines then form additional intermolecular disulfide bonds, leading to GAPDH oligomerization and aggregation (Figure 2) [57]. GAPDH aggregation is observed in diseases such as Alzheimer’s disease (Section 5).

**Table 1:** Examples of catalytic cysteine oxidation states of different GAPDH homologs.

Cysteine Oxidation to Sulfenic, Sulfinic and Sulfonic Acids or Disulfide Bond Formation					
Target Protein	Oxidant	Cys Residue	oxPTM	Influence on Enzymatic Activity	Ref.
*H. sapien* GAPDH	H_2_O_2_	Cys152	Sulfenic and sulfonic acids	Inhibition	[23]
Rabbit muscle GAPDH	H_2_O_2_	Cys150–Cys154	Intramolecular disulfide bonds	Inhibition	[58]
*A. thaliana* GAPC1	H_2_O_2_	Cys155	Sulfenic, sulfinic and sulfonic acids	Inhibition	[59]
*C.diphtheriae* GAPDH	H_2_O_2_, NaOCl	Cys153, Cys153–Cys157	Sulfonic acid, and intramolecular disulfide bond	Inhibition	[47]

In addition to intra/intermolecular disulfide bond formation, GAPDH can also form mixed disulfide bonds with LMW thiols, which serve as cellular redox buffers and contribute to the protection of proteins from H_2_O_2_ and other oxidants [60,61]. GAPDH has been reported to form mixed-disulfides with various LMW thiols, including glutathione (GSH—eukaryotes and Gram-negative bacteria), bacillithiol (BSH—Gram-positive Firmicutes), mycothiol (MSH—Actinobacteria) and coenzyme A (CoA—all organisms) (Figure 2, Table 2) [20,40,46,47,58,59,62].

Glutathionylation of GAPDH—Glutathione is a tripeptide composed of γ-glutamyl-cysteinyl -glycine, which is involved in numerous detoxification reactions and functions as a major cellular antioxidant [60,63,64]. GAPDH glutathionylation protects the catalytic cysteine from overoxidation (Figure 2), and subsequently, inactivates the glycolytic function of GAPDH (Table 2) [65,66,67,68]. GAPDH glutathionylation has been reported in different organisms including yeast, unicellular protozoan (*Plasmodium falciparum*), rabbit muscle cells and plants (*A. thaliana*), among others [48,58,59,69,70]. In vitro studies demonstrated that in the presence of H_2_O_2_, rabbit muscle GAPDH acquired two oxPTMs: glutathionylation on its catalytic cysteine (Cys150), and the formation of an intramolecular disulfide bond between Cys150 and Cys154 (non-catalytic cysteine) (Figure 2) [58]. Both redox modifications led to the reversible inactivation of GAPDH. Upon glutathionylation of GAPDH, Barinova et al. (2017) reported structural changes associated with the oxidation-induced dissociation of NAD^+^ from the active site and the decrease in GAPDH thermal stability [58]. This structural change may influence the inactivation of the enzyme and its interaction with downstream interacting partners.

In addition to H_2_O_2_-induced GAPDH glutathionylation, the catalytic cysteine of GAPDH can be glutathionylated by nitroso-glutathione (GSNO) under cellular nitrosative stress [71,72]. GSNO is considered to be the main nitric oxide (NO) reservoir within cells, and it can induce both trans-nitrosylation and glutathionylation of GAPDH and other proteins [73]. The cellular level of GSNO is controlled by prokaryotic and eukaryotic GSNO reductases [74].

Bacillithiolation and Mycothiolation of GAPDH—Most Gram-positive bacteria do not produce GSH and instead use other types of LMW thiols. Actinobacteria, e.g., *Corynebacterium diphtheriae* and *Mycobacterium tuberculosis*, use mycothiol (MSH) as their major LMW thiol [75,76]. Structurally different from GSH, MSH is composed of a cysteine residue in which the amino group is acetylated, and the carboxy group linked to glucosamine, which in turn is linked to myo-inositol [77,78]. Using shotgun liquid chromatography tandem mass spectrometry (LC-MS/MS) analysis, 26 mycothiolated proteins were identified from the proteome of *C. diphtheriae* subjected to hypochlorite (NaOCl) stress [47]. GAPDH was identified as the most abundant mycothiolated protein (0.75% of the total Cys proteome). In vitro studies using recombinant *C. diphtheriae* GAPDH demonstrated that its catalytic cysteine (Cys153) is protected from overoxidation by both mycothiolation and intramolecular disulfide bond formation in the presence of H_2_O_2_ and NaOCl. Mycothiolation of the active site cysteine was reported to inhibit its catalytic activity [47].

Other Gram-positive bacteria, e.g., *Staphylococcus aureus* and *Bacillus subtilis*, produce and use bacillithiol (BSH) as LMW thiol [20,79,80,81]. BSH structure is composed of a glycoside formed between L-cysteinyl-D-glucosamine and malic acid [79]. GAPDH was identified as the most abundant bacillithiolated protein in the proteomic study of *S. aureus* (*Sa*) subjected to NaOCl stress [46]. In the presence of H_2_O_2_ or NaOCl, *Sa*-GAPDH is bacillithiolated (in vitro) on Cys151, which reversibly inhibits its catalytic activity [46]. Molecular docking studies of BSH into the active site of GAPDH suggested that disulfide bond formation between BSH and Cys151 occurs without major conformational changes, making GAPDH the preferred bacillithiolation site in *S. aureus* under NaOCl stress [46].

CoAlation of GAPDH—Coenzyme A is an essential cofactor in all living cells. It is synthesized by an evolutionarily conserved pathway, which requires ATP, pantothenate (vitamin B5) and a cysteine residue [82]. CoA functions as a key metabolic cofactor in numerous catabolic and anabolic reactions. Recently, a novel antioxidant function of CoA was uncovered in eukaryotic and prokaryotic cells in response to oxidative and metabolic stress, and termed protein CoAlation [20,44]. Using CoA-specific monoclonal antibodies and tandem mass spectrometry, more than 1000 CoAlated proteins were identified from mammalian and prokaryotic cells subjected to oxidative or metabolic stress [20,44,83]. Under various stress conditions, GAPDH CoAlation is observed in numerous proteomic studies e.g., in mammalian cells and tissues, and in bacteria (Gram-positive *S. aureus* and *B. subtilis*, and Gram-negative *Citobacter* sp. 5-77) [20,44,62]. Tsuchiya et al. (2018) reported the reversible inactivation of *Sa*-GAPDH upon CoAlation [20]. Molecular dynamics studies proposed a possible mode of CoA binding to oxidized *Sa*-GAPDH that includes an initial binding of the CoA ADP moiety to the vacant NAD^+^-binding site (Rossmann fold), which would allow the CoA pantetheine tail to reach the catalytic Cys151 residue of GAPDH and form a mixed disulfide bond [20]. *Citobacter* sp. 5-77 GAPDH was also shown to be reversibly inactivated upon CoAlation of its catalytic Cys149 [62].

CoA has recently been reported to form a nitroso-coenzyme A (SNO-CoA) heterodimer in the presence of nitric oxide (NO) [84]. SNO-CoA and GSNO are involved in protein trans-nitrosylation [72,84]. In addition to trans-nitrosylation, GSNO can also trans-glutathionylate proteins, while the role of SNO-CoA in mediating protein CoAlation remains to be investigated. The study may shed light on the regulation of proteins (e.g., GAPDH) by SNO-CoA-mediated CoAlation during cellular nitrosative stress. SNO-CoA reductases, which reduce SNO-CoA, have been reported and proposed to play an important role in the mechanisms involving metabolic regulation by NO [84].

#### 2.2.2. Nitrosylation and Sulfhydration of GAPDH

In addition to ROS, reactive nitrogen (RNS) and sulfur (RSS) species are also produced during cellular stress, and contribute to different signaling pathways by introducing oxPTMs on proteins. Nitric oxide (NO) is a highly reactive molecule, which participates in diverse cellular signaling pathways. It causes the formation of nitrosothiol on proteins, which is termed nitrosylation (Figure 2) [85]. Protein nitrosylation can occur chemically by NO and peroxynitrite, or through trans-nitrosylation by nitrosothiols (e.g., GSNO or SNO-CoA) or other nitrosylated proteins [85,86]. The catalytic cysteine of GAPDH is a target of nitrosylation by NO and GSNO (Figure 2, Table 2), which leads to the reversible inhibition of its catalytic activity [71,73], translocation from the cytoplasm to the nucleus or mitochondria, and its contribution to different cellular processes e.g., apoptosis [8].

Similar to NO, H_2_S can also target protein cysteine thiols (sulfhydration) to form persulfide bonds (-SSH) (Figure 2). Protein sulfhydration is reported to be a more abundant PTM compared to nitrosylation within liver lysates [17]. GAPDH has been identified as a target of sulfhydration [17,87,88]. Mustafa et al. (2009) reported the sulfhydration of the catalytic cysteine of GAPDH from mouse liver lysate, which was treated with sodium hydrogen sulfide (NaHS—H_2_S donor) [17]. GAPDH sulfhydration on the catalytic cysteine was also reported in HEK293 cells treated with NaHS, which led to an increase in its activity (Table 2) [17]. In a separate study, Gao et al. (2015) observed an increase in GAPDH activity in response to elevated H_2_S production in the pancreatic cell line, MIN6, during ER stress [89]. In vitro studies revealed that inhibition of the activity of GAPDH by H_2_O_2_ and GSSG (glutathione disulfide) was reversed by H_2_S treatment [89]. Overall, the study by Gao et al. (2015) proposed that during ER stress, the control of H_2_S synthesis might regulate the activity of proteins involved in metabolic pathways (e.g., GAPDH) [89]. An in vitro study performed by Jarosz et al. (2015) reported a decrease in GAPDH activity in the presence of persulfides [88]. Furthermore, Jarosz et al. (2015) demonstrated that the activity of glutathionylated GAPDH is lower than that of the reduced enzyme, and can be partially restored upon treatment with NaHS [88]. Overall, in these studies, sulfhydration has been reported to impact the catalytic activity of GAPDH.

#### 2.2.3. Methionine Oxidation, Cysteine Disulfide Formation and GAPDH Aggregation

Human diseases (e.g., Alzheimer’s disease—Section 5) are associated with the aggregation of GAPDH due to the formation of intermolecular disulfides [57,90,91,92]. An in vitro study performed by Nakajima et al. (2007) showed that the rabbit muscle GAPDH undergoes conformational changes, and forms intermolecular disulfide bonds that lead to the aggregation of GAPDH [57]. In a later study, which focuses on the mechanism of GAPDH aggregation, Samson et al. (2014) reported that the oxidation of a highly conserved Met46 of GAPDH to methionine sulfoxide, could cause structural changes, which facilitate cysteine intermolecular disulfide formation and GAPDH aggregation [93]. In this study, the authors propose that oxidation of Met46 could initiate GAPDH structural changes, which lead to aggregation [93].

**Table 2:** Examples of S-thiolations (glutathionylation, mycothiolation, bacillithiolation and CoAlation), nitrosylation and sulfhydration on the catalytic cysteine of different GAPDH homologs. The cysteine residues shown within the table are the catalytic cysteines of the GAPDH homologs.

**Regulatory *S*-Thiolations on GAPDH**						
**Target Protein**	**Oxidant or Molecule**	**Residue**	**oxPTM**	**Influence on Enzymatic Activity**	**Recycling**	**Ref.**
*P. falciparum GAPDH*	GSSG	Cys153	Glutathion ylation	Reversible inhibition	Grx1, Trx, and plasmo- redoxin	[70]
Rabbit muscle GAPDH	H_2_O_2_ (+GSH)	Cys150	Glutathionylation	Reversible inhibition	Excess GSH, and Trx	[58]
*A. thaliana* GAPC1	GSSG, and H_2_O_2_ (>+GSH)	Cys155 *	Glutathionylation	Reversible inhibition	GrxC1, and Trx	[59]
*A. thaliana* A4-GAPDH	GSSG, and H_2_O_2_ (+GSH)	Cys149	Glutathionylation	Reversible inhibition	Grx1, and Grx3	[94,95]
*H. sapiens* GAPDH	H_2_O_2_ (+GSH)	Cys152	Glutathionylation	Reversible inhibition	DTT	[23]
*C. diphtheriae* GAPDH	H_2_O_2_ (+MSH), and NaOCl (+MSH)	Cys153	Mycothiolation	Reversible inhibition	Mrx1, and Trx	[47]
*S. aureus* GAPDH	H_2_O_2_ (+BSH), and NaOCl (+BSH)	Cys151	Bacillithiolation	Reversible inhibition	Brx	[46]
*S. aureus* GAPDH1	CoASSCoA	Cys151	CoAlation	Reversible inhibition	DTT	[20]
*Citobacter sp*. 5-77 GAPDH	CoASSCoA, NaOCl (+CoA), and H_2_O_2_ (+CoA)	Cys149	CoAlation	Reversible inhibition	Excess DTT, GSH, and CoA	[62]
**Nitrosylation and Sulfhydration of GAPDH**						
**Target Protein**	**Oxidant or Molecule**	**Residue**	**oxPTM**	**Influence on Enzymatic Activity**	**Recycling**	**Ref.**
*A. thaliana* GAPC1	GSNO	Cys155 *	Nitrosylation	Reversible inhibition	GSH	[72]
GAPDH (SH-Sy5Y cell extract)	SNO-Trx1	Cys247	Nitrosylation	Reversible inhibition	Reduced Trx1	[96,97]
GAPDH (HEK293 extract)	H_2_S	Cys152	Sulfhydration	Activity increase	DTT	[17]

* The catalytic cysteine of *A. thaliana* GAPC1 is at position 155; however, it is referred to as Cys149 in the cited publications, to be consistent with the first solved GAPDH structure (PDB: 2DBV) in which the catalytic cysteine residue is numbered 149.

## 3. Decoding of the GAPDH Redox Communication

Diverse proteins have been identified, which recognize and interact with redox-modified GAPDH, regulating its glycolytic and non-glycolytic (moonlighting) functions. In this section, we describe these GAPDH readers, particularly focusing on those proteins, which decode and transduce the redox information, or amplify it via the attachment of LMW thiols (e.g., for the case of sulfenylated GAPDH).

### 3.1. Readers of Sulfenylated and S-Thiolated GAPDH

The catalytic cysteine of GAPDH plays a crucial role in defining its activity, interaction with partners, and its role in the regulation of cellular processes. A study performed in *Schizosaccharomyces pombe* showed that under H_2_O_2_-induced stress, redox-modified GAPDH participates in phospho-relay signaling [98]. *S. pombe* phosphorelay signaling is activated by external stimuli (e.g., H_2_O_2_) and is composed of a two-component system: (a) the multi-step phosphorelay (sensor kinase, phosphotransferase and response regulator, Mcs4), which transmits H_2_O_2_ stress signal to (b) the mitogen-activated protein kinase (MAPK) cascade [99,100]. Mcs4 (response regulator) activates MAPK cascade. In response to H_2_O_2_-induced stress, the catalytic cysteine (Cys152) of *S. pombe* GAPDH (Tdh1) is transiently oxidized, and in turn, enhances the association of Tdh1 with Mcs4 [98]. Therefore, in *S. pombe*, oxidation of GAPDH may provide additional input signal, and promote peroxide stress signaling through the multistep phosphrelay system [98]. In addition, the *tdh1* null mutant cells have a reduced H_2_O_2_-stress response through phosphorelay signaling, compared to wild-type cells [98].

In another study, the H_2_O_2_-induced modification on the catalytic cysteine of mammalian GAPDH is reported to participate in the H_2_O_2_-dependent activation of mammalian phospholipase D2 (PLD2) in PC12 cells, which mediates an anti-apoptotic effect during oxidative stress [101,102]. PLD2 catalyzes the hydrolysis of the phosphodiester bond of phosphatidylcholine, which generates phosphatidic acid, a lipid second messenger [103]. The study shows that the H_2_O_2_-modified catalytic cysteine of GAPDH acts as a positive regulator of PLD2 [101].

In both examples mentioned above, oxidation of GAPDH enhances its recognition and binding to different types of GAPDH readers. Alternatively, GAPDH readers may also recognize the *S*-thiolated form of GAPDH. For example, CoAlation of GAPDH introduces a bulky molecule (pantetheine tail and ADP moiety) within the GAPDH active site [20]. The CoA ADP moiety could be recognized by Rossmann fold-containing proteins (readers), which can facilitate the interaction between GAPDH and the reader protein. The interaction between these readers and GAPDH could potentially lead to the regulation of different cellular processes. Similarly, the *S*-mycothiolation or *S*-bacillithiolation of GAPDH may possibly enhance binding of proteins that recognize these bulky molecules, which are covalently bound to the GAPDH catalytic cysteine residue.

### 3.2. Readers of Nitrosylated GAPDH

#### 3.2.1. Nuclear Transportation and Cellular Apoptosis

Nitrosylation of mammalian GAPDH has been associated with triggering an apoptotic cascade, which is initiated in the cytoplasm. When cells are initially exposed to a stressor, and the level of nitric oxide (NO) within cells becomes slightly higher than physiological concentrations, GOSPEL (GAPDH’s Competitor of Siah Protein Enhances Life) becomes nitrosylated on Cys47, binds to GAPDH, and competes with Siah1, an E3 ubiquitin ligase, to retain GAPDH in the cytoplasm [104]. The binding of GOSPEL to GAPDH maintains cellular homeostasis under nitrosative stress, and prevents the triggering of a cellular apoptotic cascade [104]. However, an increase in the level of nitrosative stress beyond a certain threshold leads to the nitrosylation of the catalytic Cys150 of rat GAPDH (forming SNO-GAPDH) [8]. SNO-GAPDH is recognized by Siah1, and their interaction is induced by cell stressors which augment nitric oxide production. Siah1 marks proteins for proteasomal degradation by ubiquitinating the target proteins. It contains a nuclear localization signal, which mediates the translocation of the SNO-GAPDH-Siah1 complex to the nucleus [8]. The nuclear complex interacts with various target proteins and regulates their function by e.g., ubiquitination, acetylation or trans-nitrosylation, which lead to apoptosis.

Once in the nucleus, the stabilized Siah1 causes ubiquitination and subsequent degradation of the nuclear co-repressor (N-Cor), which contributes to cellular apoptosis. On the other hand, nuclear SNO-GAPDH directly interacts with the acetyltransferase p300/CBP (CREB-binding protein) [105]. Their binding initiates the auto-acetylation and subsequent activation of p300/CBP, which acetylates downstream targets (including tumor suppressor p53) that contribute to cellular apoptosis [105]. Once activated, p53 induces apoptosis by trans-activating numerous downstream pro-apoptotic genes, e.g., mainly the expression of p53-up-regulated modulator of apoptosis (PUMA) [105,106]. Nuclear SNO-GAPDH can also trans-nitrosylate and alter the function of several nuclear proteins e.g., deacetylase sirtuin-1 (SIRT1), histone deacetylase-2 (HDAC2) and DNA-activated protein kinase (DNA-PK) [107].

An alternative mechanism for the binding of oxidized GAPDH to Siah1 has been reported [108]. It involves the apoptosis signal-regulating kinase 1 (ASK1), which is activated in response to different cellular stresses [109,110]. Tristan et al. (2015) reported that ASK1 could enhance the interaction between GAPDH and Siah1 by phosphorylating Siah1. This in turn leads to the activation of GAPDH-Siah1 nuclear signaling cascade, followed by the activation of acetyltransferase p300, and the induction of cellular apoptosis [108].

In addition to the induction of an apoptotic cascade, nuclear GAPDH also participates in DNA repair, DNA replication and telomere maintenance [6,7].

#### 3.2.2. GAPDH Translocation to the Mitochondria

Upon exposure to stress, the level of mitochondrial GAPDH has been reported to increase [111,112]. This may indicate the functional requirement of GAPDH within the mitochondria during cellular stress. Kohr et al. (2014) show that SNO-GAPDH is able to translocate to the matrix of mitochondria isolated from ischemically preconditioned mouse heart [112]. Mitochondrial ischemic preconditioning has been reported to increase the level of nitrosylated mitochondrial proteins [113]. Several mitochondrial proteins, e.g., voltage-dependent anion channel (VDAC1—outer mitochondrial membrane), Hsp60 and acetyl-CoA acetyltransferase (ACAT1), were shown to interact with and to be transnitrosylated by SNO-GAPDH [112]. Therefore, future studies might shed light on the role of SNO-GAPDH in regulating mitochondrial function by trans-nitrosylating specific mitochondrial proteins and regulating their function.

### 3.3. Readers of Sulfhydrated GAPDH

Mir et al. (2014) reported that interleukin-1β causes the degradation of postsynaptic density 95 (PSD95) in a H_2_S-dependent manner [114]. PSD95 is a scaffolding protein, which plays an important role in synapse maturation, stability, and plasticity [115]. In the presence of IL-1β, the expression of cystathionine beta-synthase (CBS) increases, which leads to the production of H_2_S. Upon exposure to H_2_S, GAPDH is reported to become sulfhydrated and binds to Siah1 [114]. In complex with sulfhydrated GAPDH, Siah1 interacts with and ubiquitinates PSD95, which results in PSD95 degradation and synapse loss within the brain [114].

## 4. Erasers of Redox-Associated Modifications

**Figure 3:**
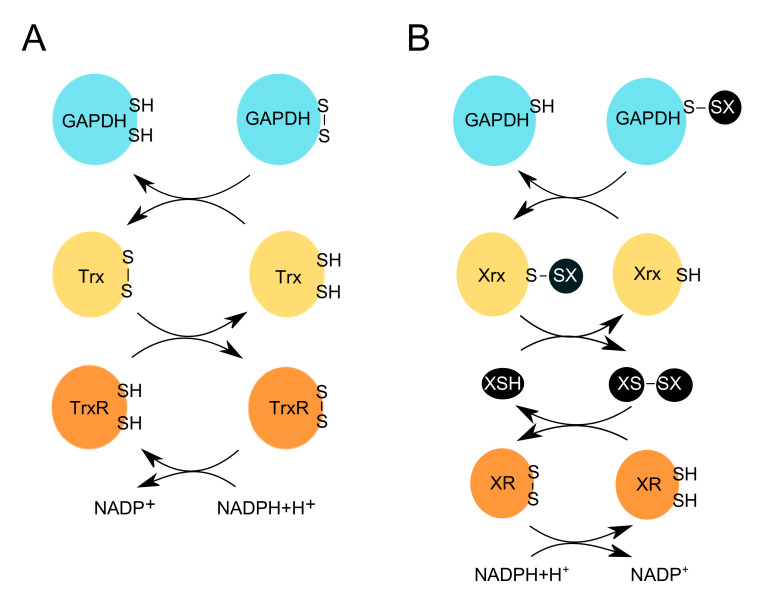
Redox erasers restore the reduced and active form of GAPDH. OxPTMs introduced on GAPDH by the redox writers (e.g., H_2_O_2_) and editors (e.g., XSH, where X can be glutathione, or other LMW thiols, e.g., mycothiol, bacillithiol or coenzyme A), are removed by redox erasers e.g., (**A**) thioredoxin (Trx), or (**B**) glutaredoxin (Grx), respectively. (**A**) Trx reduces the disulfide bond on GAPDH. This leads to the release of the reduced form of GAPDH, and the oxidized form of Trx. The latter is then reduced by TrxR, which uses NADPH+H^+^ to restore its reduced form. (**B**) Redoxins (Xrx) e.g., glutaredoxins (Grx), reduce the glutathionylated form of GAPDH. This leads to the release of the reduced form of GAPDH, and the subsequent glutathionylation of Grx. Another molecule of e.g., GSH, can then reduce Grx, and in turn, form glutathione disulfide (GSSG). The latter is then reduced by glutathione reductase (GR), which uses NADPH+H^+^ to restore its reduced form.

“Redox erasers” remove redox-associated modifications and restore the reduced form of modified proteins. In turn, they network with other antioxidant enzymes/molecules to restore their own activity by using NADPH+H^+^ as the final electron donor. In this section, we will focus on the redox regulation of GAPDH by the thioredoxin pathway and the LMW thiol pathways (Figure 3).

### 4.1. Key Features of Cellular Redox Erasers

Redox-sensitive cysteines participate in diverse cellular signaling and metabolic pathways. The activity and coordination of these cysteines depend on molecular thiol switches such as thioredoxin (Trx) and glutaredoxin (Grx) (Figure 3). The thioredoxin pathway takes part in the cellular antioxidant response by reducing disulfide bonds formed on target proteins. This pathway catalyzes the electron transfer from NADPH+H^+^, through the flavoenzyme thioredoxin reductase (TrxR) to Trx. The latter can control numerous signaling pathways by interacting with and transferring electrons to different proteins, which form disulfide bonds when oxidized (Figure 3) [116,117,118,119]. Another reducing enzyme is glutaredoxin (Grx), which can deglutathionylate proteins via the monothiol mechanism.

*S*-glutathionylation is reversed by Grx, which can reduce the mixed disulfide bond and subsequently, become itself glutathionylated. The second mixed disulfide between GSH and Grx is then attacked by another GSH molecule resulting in the release of the reduced form of Grx and a glutathione disulfide molecule (GSSG). The GSSG is reduced by glutathione reductase (GR), which uses NADPH+H^+^ as its final electron donor (Figure 3) [120,121].

### 4.2. Erasers of GAPDH Redox Modifications

#### 4.2.1. Erasers of Intra- and Intermolecular Disulfide Bonds

The redox-induced formation of intra- and intermolecular disulfide bonds has been reported for GAPDH (Table 1) [93,122,123,124]. Different reducing pathways participate in recycling the oxidized GAPDH, and in recovering its catalytic activity (Figure 3, Table 1). Landino et al. (2014) showed that the H_2_O_2_-induced oxidation of GAPDH resulted in the formation of disulfide bonds, which were reduced by the Trx pathway [123]. Interestingly, within the same study, they showed a possible thiol-disulfide exchange between tubulin and GAPDH, making tubulin a potential redox eraser of oxidized GAPDH [123]. Future studies on understanding the detailed thiol-disulfide exchange mechanism between tubulin and oxidized GAPDH may shed light on how tubulin could potentially function as a redox eraser. Important to note is that tubulin is an abundant protein in neuronal cells, which interacts with different microtubule-associated proteins (MAP) such as Tau and MAP2 [125]. These proteins have been reported to be redox-sensitive and can form disulfide bonds that reduce their microtubule binding ability. Trx and Grx are reported to reduce these disulfides and restore their function [126,127,128].

#### 4.2.2. Erasers of GAPDH *S*-Thiolation

Redox erasers, such as thioredoxins, glutaredoxins, mycoredoxins, bacilliredoxins and CoAredoxins use thiol-disulfide exchange mechanisms to regulate proteins with diverse cellular functions (Figure 3 and Table 2). The mechanisms of GAPDH deglutathionylation have been studied in numerous organisms, such as yeast, unicellular protozoans (*P. falciparum*), plants (*A. thaliana*) and animals [48,59,69,70,94,129]. The catalytic activity of the glutathionylated cytosolic *A. thaliana* GAPDH (GapC1 isoform) recovers upon treatment with cytosolic Grx [59]. The latter deglutathionylates GAPDH using the GSH-dependent monothiol mechanism, and restores its catalytic activity. Interestingly, although Trx enzymes have been reported to reduce sulfenic acids and disulfide bonds, recent studies have revealed that they can also participate in the deglutathionylation of GapC1, but less efficiently compared to Grx [59]. The activity of glutathionylated *P. falciparum* GAPDH is also restored upon treatment with both Grx1 and Trx1, in addition to the *P. falciparum*-specific plasmoredoxin [70]. The glutathionylated *A. thaliana* chloroplastic GAPDH (A4-GAPDH isoform) was deglutathionylated by Grx1 (cytosolic isoform) and Grx3 (chloroplastic isoform) [94], while glutathionylated yeast GAPDH (Tdh3) was deglutathionylated by Trx1, Trx2 [69], and the yeast mitochondrial matrix Grx5 [130]. Interestingly, the glutathionylated rabbit muscle GAPDH was reported to be deglutathionylated non-enzymatically in the presence of GSH or enzymatically by Trx [58]. The latter only partially reactivated GAPDH [58]. Overall, the mechanisms of protein deglutathionylation depend on the organism, subcellular localization of GAPDH, and the availability and specificity of “redox eraser” isoforms.

Demycothiolation of GAPDH was found to involve mycoredoxin-1 (Mrx1) and thioredoxin. The latter was considered to be less efficient in demycothiolating GAPDH compared to Mrx1 [47]. Bacillithiol-modified *S. aureus* GAPDH is shown to be debacillithiolated by bacilliredoxin, which uses BSH, bacillithiol disulfide reductase and NADPH+H^+^ to restore its activity [46,131]. In contrast, the mechanism of protein deCoAlation is not well understood. Analysis of mammalian and bacterial cells exposed to different oxidative and metabolic stresses revealed extensive protein CoAlation, which was rapidly reversed after the removal of oxidizing agents [20,44]. This suggests the presence of a CoAredoxin activity, which remains to be further explored. A study performed by Tsuji et al. (2019) showed that *Citrobacter* sp. S-77 GAPDH can be in vitro deCoAlated non-enzymatically by excess of GSH, and less efficiently by excess of CoA [62].

#### 4.2.3. Erasers of GAPDH Nitrosylation and Sulfhydration

A recent study showed that human GAPDH could also be trans-nitrosylated by the nitrosylated form of thioredoxin-1 (SNO-Trx1). Interestingly, the reduced form of Trx1 was proposed to denitrosylate SNO-GAPDH, which restored its catalytic activity [96,97,132]. Therefore, Trx1 may play an important role by regulating the apoptotic function of GAPDH via nitrosylation/denitrosylation mechanisms. A study performed on the *A. thaliana* cytosolic GAPDH (GapC1) showed that SNO-GAPDH is denitrosylated non-enzymatically in the presence of excess GSH [72]. This GSH-dependent denitrosylation of GAPDH fully recovered its catalytic activity, without glutathionylating GAPDH. Further studies demonstrated that the GapC1 denitrosylation was directly linked to the cellular ratio between glutathione and nitrosoglutathione ([GSH]/[GSNO]) [72].

Knowledge of the desulfhydration mechanism of GAPDH is emerging. Ju et al. (2016) showed that thioredoxin can desulfhydrate proteins. Cells overexpressing Trx1 showed a decrease in the level of sulfhydrated proteins, which included GAPDH. Mutation of the Trx catalytic cysteine (Cys32) abolished its GAPDH desulfhydration function [133]. This may suggest that Trx can have an important role in the regulation of endogenous sulfhydrated proteins, which have important cellular signaling and metabolic functions.

Overall, diverse redox-induced PTMs of GAPDH are temporally and spatially introduced to coordinate its activity, subcellular localization, and function via the interaction with redox readers and the formation of multi-enzyme regulatory complexes. The function of the redox erasers is critical for restoring the reduced form of GAPDH and thus supporting its glycolytic and moonlighting functions.

## 5. Human Pathologies Associated with Dysregulated GAPDH Function

The dysregulation of GAPDH function by oxPTMs has been associated with various human pathologies, including neurodegeneration and metabolic disorders. Such aberrations in GAPDH function include, among others, altered glycolytic activity, involvement in amyloidogenesis and induction of apoptosis. Therefore, GAPDH is an attractive target for the development of diagnostic and therapeutic approaches. This section briefly summarizes the roles GAPDH plays in neurodegeneration and metabolic disorders, as well as therapeutic compounds that target redox-modified GAPDH.

### 5.1. GAPDH in Neurodegeneration

Neurodegenerative diseases are pathological conditions in which the nervous system progressively deteriorates, eventually leading to neuronal death. Aberrant GAPDH function has been associated with different neurodegenerative pathologies, including Alzheimer’s disease (AD), Parkinson’s disease (PD) and Huntington’s disease (HD). It is well known that oxidative stress is linked to the progression of different neurodegenerative pathologies. In vitro studies of GAPDH oxidation were shown to induce structural changes, and intermolecular disulfide bond formation, which cause aggregation of this enzyme [57,93]. GAPDH aggregates were observed in post-mortem brain extracts of AD patients [90,134], while aged brains of AD transgenic mice contain insoluble disulfide-bonded form of GAPDH, which does not occur in control mice [90]. Oxidized GAPDH has also been directly linked to AD progression through its association with amyloidogenic proteins e.g., amyloid-β (Aβ) and Tau. The progression of AD is linked to the accumulation of extracellular Aβ deposits in the brain, which are induced by oxidative stress and eventually lead to the formation of amyloid plaques and neuronal death. Oxidized GAPDH is capable of forming a complex with soluble Aβ and accelerating its aggregation [135]. On the contrary, the reduced form of GAPDH is unable to form a stable complex with Aβ. Studies on the post-mortem AD brain tissues showed that GAPDH co-localizes with both plaque-like structures and neurofibrillary tangles in AD patient brains [134]. Similarly, in PD, GAPDH was found to promote the aggregation of the amyloidogenic protein, α-synuclein, into Lewy body-like inclusions [136].

Different oxPTMs on GAPDH, e.g., nitrosylation and glutathionylation, have been reported in AD. Studies have reported a 7-fold and 2-fold increase in the level of glutathionylated and nitrosylated GAPDH, respectively, in the brains of AD patients, compared to control individuals [137,138]. Both these modifications decrease the glycolytic function of GAPDH, and may contribute to the loss of neuronal function in AD brains. Furthermore, oxPTMs, e.g., nitrosylation, may promote pathological nuclear targeting of GAPDH. Indeed, nuclear GAPDH was observed in postmortem brain tissues of AD [90,139] and PD [140] patients, and HD transgenic mice [141], indicating that nuclear targeting of the enzyme may be a common pathological feature in different neurodegenerative pathologies. Interestingly, the nuclear GAPDH in samples of AD brains [90,139] was found in an aggregated form, suggesting that nuclear translocation and amyloidogenesis of GAPDH may operate synergistically in neurodegeneration. Studies in AD have also shown that the neurotoxic Aβ is capable of inducing disulfide bond formation on GAPDH, which can promote its nuclear translocation and pro-apoptotic function [90,139].

### 5.2. GAPDH in Metabolic Disorders

Dysregulated GAPDH function is also implicated in metabolic disorders, particularly diabetes. Hyperglycemia is a common diabetic condition that causes vascular pathology in tissues throughout the body and associates with superoxide generation [142]. Hyperglycemia-induced superoxide production has been linked to the inhibition of GAPDH glycolytic activity, resulting in the activation of protein kinase C signaling, hexosamine biosynthetic pathway, and generation of advanced glycation end products (AGEs) [143,144,145,146]. Redox-induced PTMs of GAPDH were reported in several studies on diabetic retinopathy, a disease resulting from chronic hyperglycemia. Hyperglycemic conditions were shown to induce nuclear translocation of GAPDH and consequent apoptosis of human retinal Müller and pericyte cells [147,148,149,150,151], both of which undergo cell death in diabetic retinopathy [150]. Importantly, this apoptotic cascade is dependent on GAPDH-Siah-1 complex formation [147,148], which is known to be promoted by GAPDH nitrosylation [8]. These findings are supported by the demonstration that R-(−)-Deprenyl, a drug that prevents S-nitrosylation of GAPDH, can reduce nuclear GAPDH accumulation and associated apoptosis in human Müller cells [149]. Hyperglycemia-induced superoxide and nitric oxide production may also lead to the accumulation of peroxynitrite and protein nitration. Indeed, increased nitration of GAPDH has been detected in the retina of diabetic rats [152].

### 5.3. Therapeutic Targeting of Redox-Modified GAPDH

The development of compounds capable of counteracting the aberrant effects of oxPTMs on GAPDH function in human pathologies has emerged as a promising direction of research. Several compounds have been already developed, including the PD drug R-(−)-Deprenyl and its derivative TCH346, which bind to GAPDH, preventing its *S*-nitrosylation and associated nuclear translocation [153]. Notably, R-(−)-Deprenyl can prevent cell death induced by etoposide, a process associated with nuclear translocation of GAPDH [153]. Similarly, a synthetic compound named AXP3009, binds to the redox-sensitive Cys152 of *Plasmodium falciparum* GAPDH, and reduces nuclear translocation of GAPDH and associated cellular apoptosis [154,155].

Although R-(−)-Deprenyl is a potent inhibitor of GAPDH nuclear translocation, it is not capable of averting GAPDH aggregation, highlighting the multifactorial nature of redox-induced GAPDH dysregulation in pathologies. In contrast, the naturally occurring compound piceatannol was reported to prevent both nuclear translocation and aggregation of GAPDH [156]. Piceatannol was demonstrated to covalently bind the catalytic cysteine of GAPDH and preclude GAPDH nuclear translocation and apoptosis in cells exposed to oxidative stress, while also preventing formation of intermolecular disulfide bonds and associated GAPDH aggregation [156]. Several small molecule compounds of plant origin have been recently shown to inhibit oxidative stress-induced GAPDH aggregation [157], by binding to the NAD^+^-binding site of GAPDH. Among these, the compounds RX409 and RX426 were protective against cultured neuroblastoma cell death induced by hydrogen peroxide, as well as murine brain injury and associated motor impairments induced by malonic acid injection; in both settings, protection was accompanied by reduced GAPDH aggregation [157].

## 6. Conclusions

Different metabolites produced during cellular stress (metabolic and oxidative stress) contribute to the redox regulation of numerous proteins and signaling pathways, which can direct the cellular machinery toward cell survival or death. By introducing redox modifications on target proteins, these metabolites may alter the conformation, subcellular localization, and function of the target protein, and modulate its downstream interactions with partners and signaling complexes. GAPDH is an example of a well-studied protein, which is redox regulated. Its redox-sensitive catalytic cysteine is considered to be a “hub” of different oxPTMs, which direct GAPDH toward different cellular compartments and numerous non-glycolytic functions. These oxPTMs can enhance or diminish the interaction of GAPDH with various downstream partners (oxGAPDH readers) in different cellular organelles, where it can participate either in cell survival or death. The removal of oxPTMs involves different cellular recycling pathways (redox erasers), which reduce the GAPDH catalytic cysteine and restore its glycolytic function. This highlights the complexity of the redox regulatory mechanisms within cells. The dysregulation of GAPDH during oxidative stress has been associated with human pathologies, including neurodegenerative diseases (AD, PD and HD), and metabolic disorders, among others. Given the association of oxPTMs on GAPDH with different diseases, there are still many unknown mechanisms/interactions of GAPDH that remain to be explored and understood. Future studies on deciphering the role of regulatory interactions and oxPTMs of GAPDH in health and disease will provide the insight and direction needed for advancing the development of novel diagnostic and therapeutic approaches targeting GAPDH.
